# Supplementary figures and images for: Repression of PRMT activities sensitize homologous recombination-proficient ovarian and breast cancer cells to PARP inhibitor treatment
Source: bioRxiv. 2025 Aug 11:2024.05.21.595159. Originally published 2024 May 21. Preprint. [Version 4] doi: 10.1101/2024.05.21.595159 (PMC11142138; doi:10.1101/2024.05.21.595159)

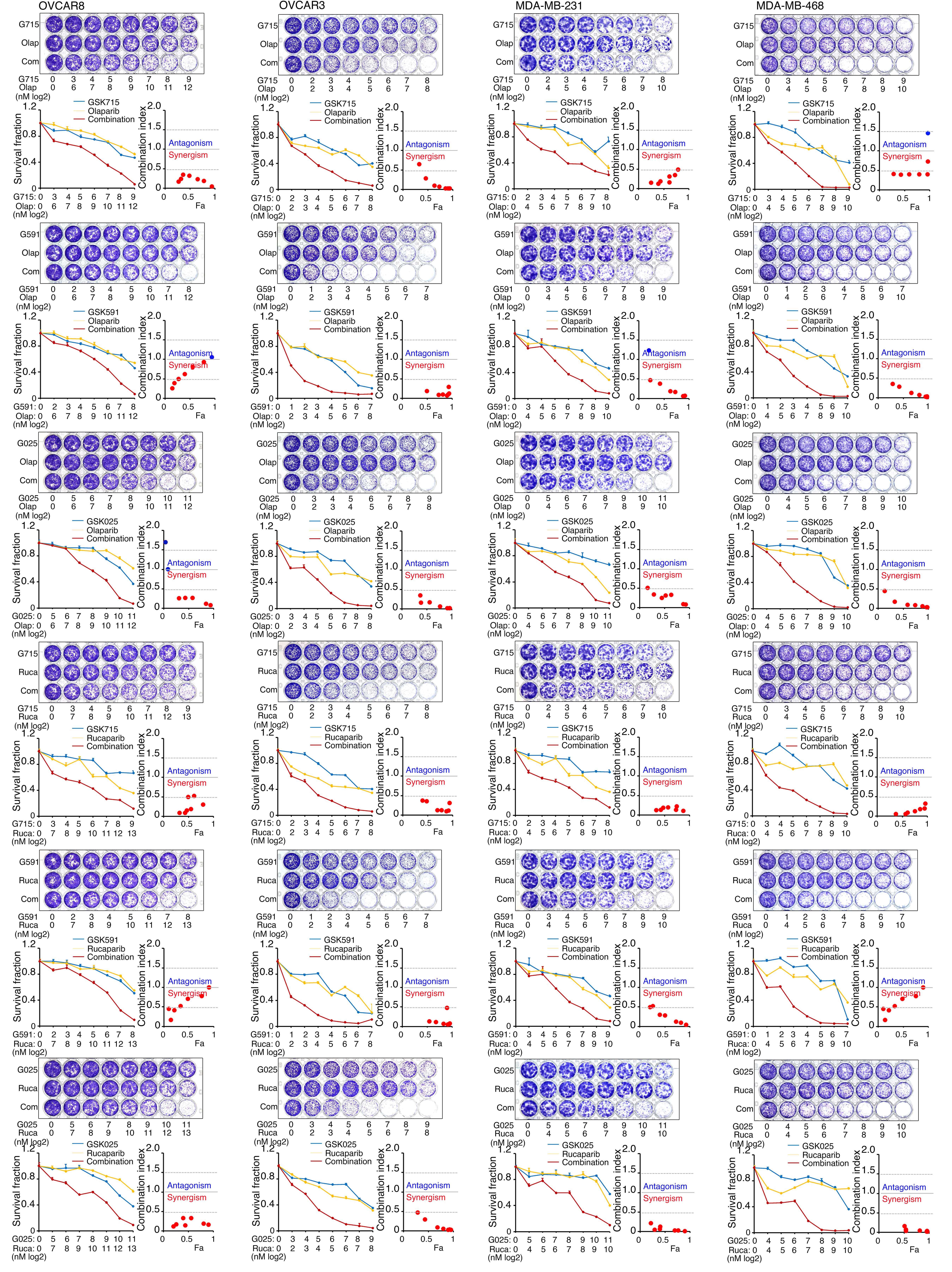

Supplement: Supplement 1 — Figure S1. The sensitivity of cancer cells to PARPi alone, PRMTi alone, and a PARPi and PRMTi combination in a panel of HGSOC and TNBC cell lines. For each treatment, the upper panel shows crystal violet staining of a colony formation assay; the lower left panel shows a quantified survival fraction; and the lower right panel shows the CI values. Fa, fraction affected. [file media-1.jpg]

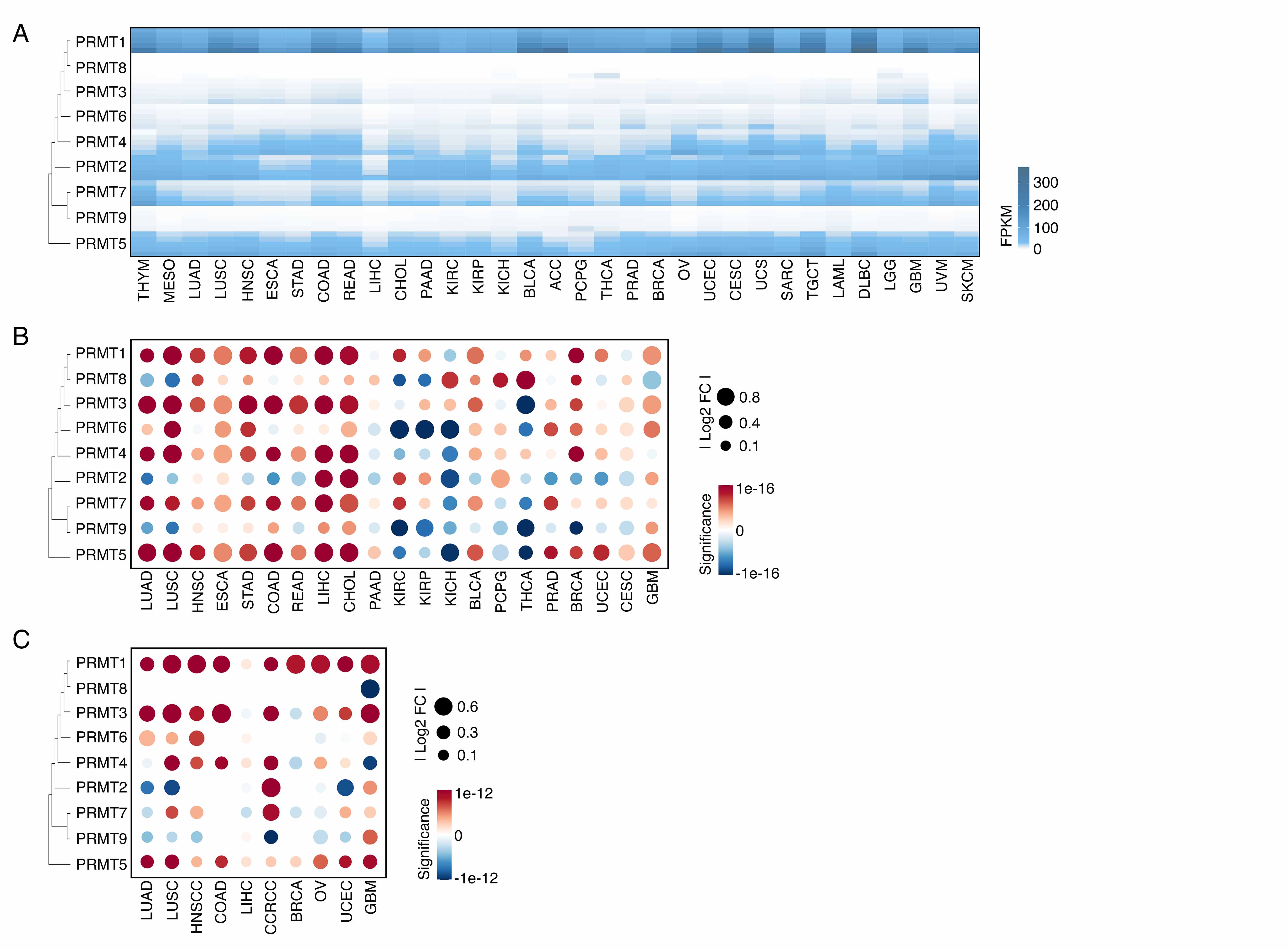

Supplement: Supplement 2 — A. The heatmap of mRNA expression levels of PRMT family across the TCGA tumor samples. The intensity of heatmap color indicates the FPKM value for each percentile (25th, 50th, 75th, and 90th) of an individual PRMT gene in a given cancer type. B. The mRNA expression levels of the PRMT family in tumors compared with corresponding controls in the TCGA cohorts. Red and blue indicate up- and down-regulation, respectively. C. The protein expression levels of the PRMT family in tumors compared with corresponding controls in the CPTAC cohorts. Red and blue indicate up- and down-regulation, respectively. [file media-2.jpg]

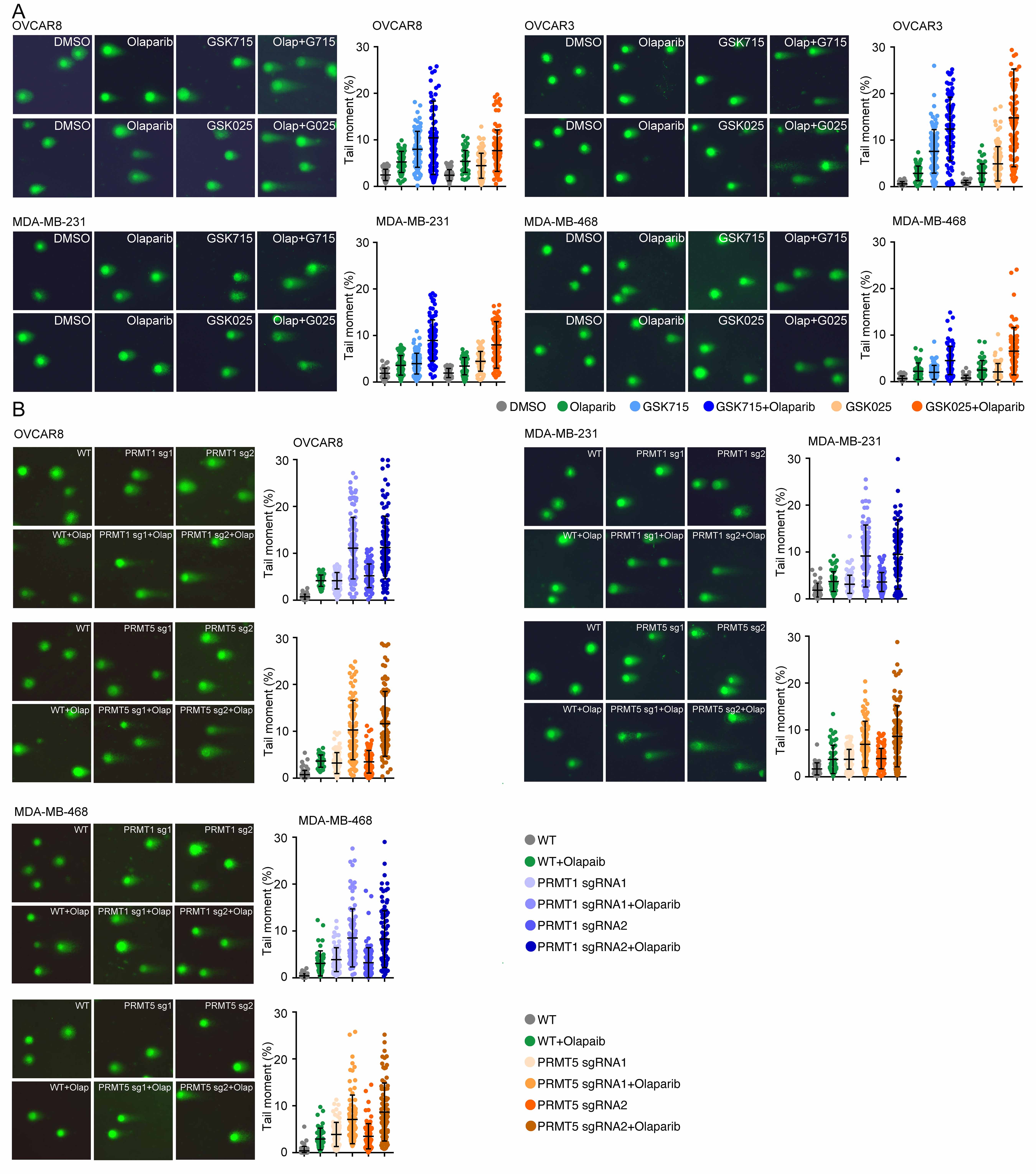

Supplement: Supplement 3 — A. Left panels: A comet assay was used to measure DNA damage in OVCAR8, OVCAR3, MDA-MB-231, and MDA-MB-468 cell lines treated with DMSO, olaparib, GSK3368715, GSK3235025, or combinations. Scale bars, 10 μm. Right panels: The extent of DNA damage was quantified using the tail moment in comet assays of OVCAR8, OVCAR3, MDA-MB-231, and MDA-MB-468 cell lines treated with DMSO, olaparib, GSK3368715, GSK3235025, or combinations. Data are presented as means ± SDs, *p < 0.05 determined by two-tailed Student’s t tests. B. Left panels: A comet assay was used to measure olaparib treatment-induced DNA damage in OVCAR8, MDA-MB-231, and MDA-MB-468 cell lines in which PRMT1 and PRMT5 were independently knocked out using lentiviral CRISPR/Cas9. Scale bars, 10 μm. Right panels: The extent of olaparib treatment-induced DNA damage was quantified using the tail moment in comet assays of OVCAR8, MDA-MB-231, and MDA-MB-468 cell lines in which PRMT1 and PRMT5 were independently knocked out using lentiviral CRISPR/Cas9. Data are presented as means ± SDs, *p < 0.05 determined by two-tailed Student’s t tests. [file media-3.jpg]

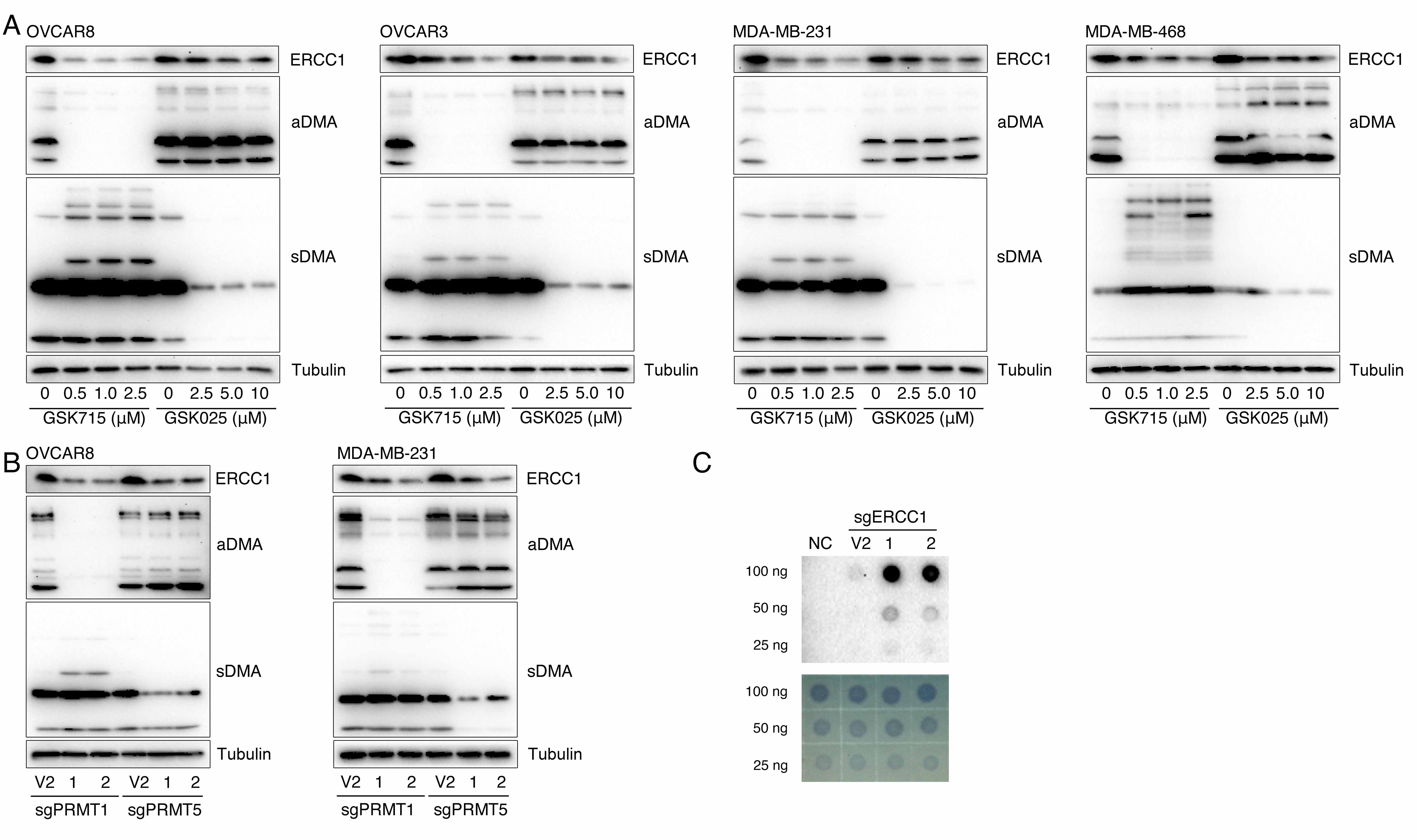

Supplement: Supplement 4 — A. The expression levels of aDMA and sDMA in cancer cell lines in which PRMTs were inhibited by chemical compounds. The expression levels of aDMA and sDMA in cancer in OVCAR8, OVCAR3, MDA-MB-231, and MDA-MB-468 cell lines treated with GSK3368715 or GSK3235025. B. The expression levels of aDMA and sDMA in cancer cell lines in which PRMTs were inhibited by genetic approach. The expression levels of aDMA and sDMA in cancer in OVCAR8 and MDA-MB-231cell lines in which PRMT1 or PRMT5 was knocked out by CRISPR/Cas9. C. Dot blots of CPD levels in MDA-MB-231 cells in which ERCC1 was knocked out using lentiviral CRISPR/Cas9. Methylene blue staining was used as the loading control. [file media-4.jpg]

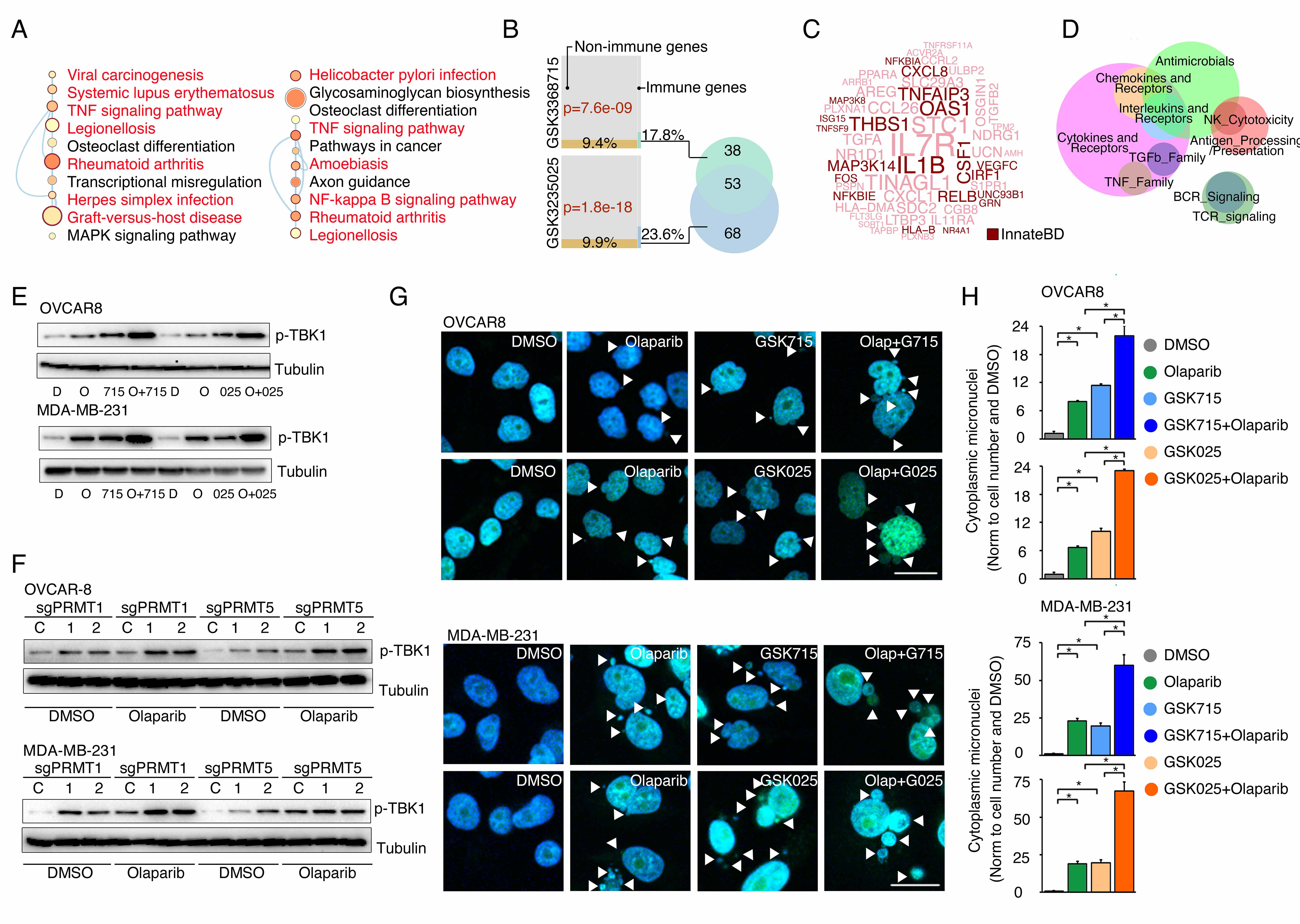

Supplement: Supplement 5 — A. The top 10 KEGG pathways in which genes upregulated by GSK3368715 (left) and GSK3235025 (right) treatments were significantly enriched. Circle size indicates fold enrichment. Lines indicate numbers of shared genes between pathways. The immune-related pathways are highlighted in red. B. Mosaic plots show percentages of genes upregulated by GSK3368715 (upper) and GSK3235025 (lower) treatments. Right: immune-related genes, left: non-immune-related genes. Significantly more immune-related genes, compared with non-immune related genes, were upregulated by PRMT inhibition (GSK3368715:17.8% vs 9.4%; GSK3235025: 23.6% vs 9.9%). C. Word cloud revealing the common immune-related genes upregulated by GSK3368715 and GSK3235025 treatments. 37.7% are associated with innate immune responses (dark red). Text size indicates meta-fold changes by both PRMTis. D. Scaled Venn diagram showing the functional families among the common upregulated immune-related genes. E. Western blot analysis of p-TBK1 expression in OVCAR8 (upper panel) and MDA-MB-231 (lower panel) cells treated with DMSO, olaparib (5 μM), GSK3368715 (OVCAR8, 5 μM and MDA-MB-231, 2.5 μM), GSK3235025 (5 μM) or olaparib (5 μM) combined independently with GSK3368715 (OVCAR8, 5 μM and MDA-MB-231, 2.5μM) and GSK3235025 (5 μM) for 96 h. F. Western blot analysis of p-TBK1 expression in OVCAR8 (upper panel) and MDA-MB-231 (lower panel) cells in which PRMT1 and PRMT5 were independently knocked out using lentiviral CRISPR/Cas9. The cells were treated with olaparib (5 μM, 96 h) or DMSO. G. Representative cytoplasmic micronuclei stained by PicoGreen in OVCAR8 (upper panel) and MDA-MB-231 (lower panel) cells treated with DMSO, olaparib (5 μM), GSK3368715 (OVCAR8, 5 μM and MDA-MB-231, 2.5 μM), GSK3235025 (5 μM), and olaparib (5μM) combined independently with GSK3368715 (OVCAR8, 5 μM and MDA-MB-231, 2.5 μM) and GSK3235025 (5 μM). White arrows: micronuclei. Scale bar: 10 μm. H. Quantification of micronuclei from G. Micronuclei number p [file media-5.jpg]

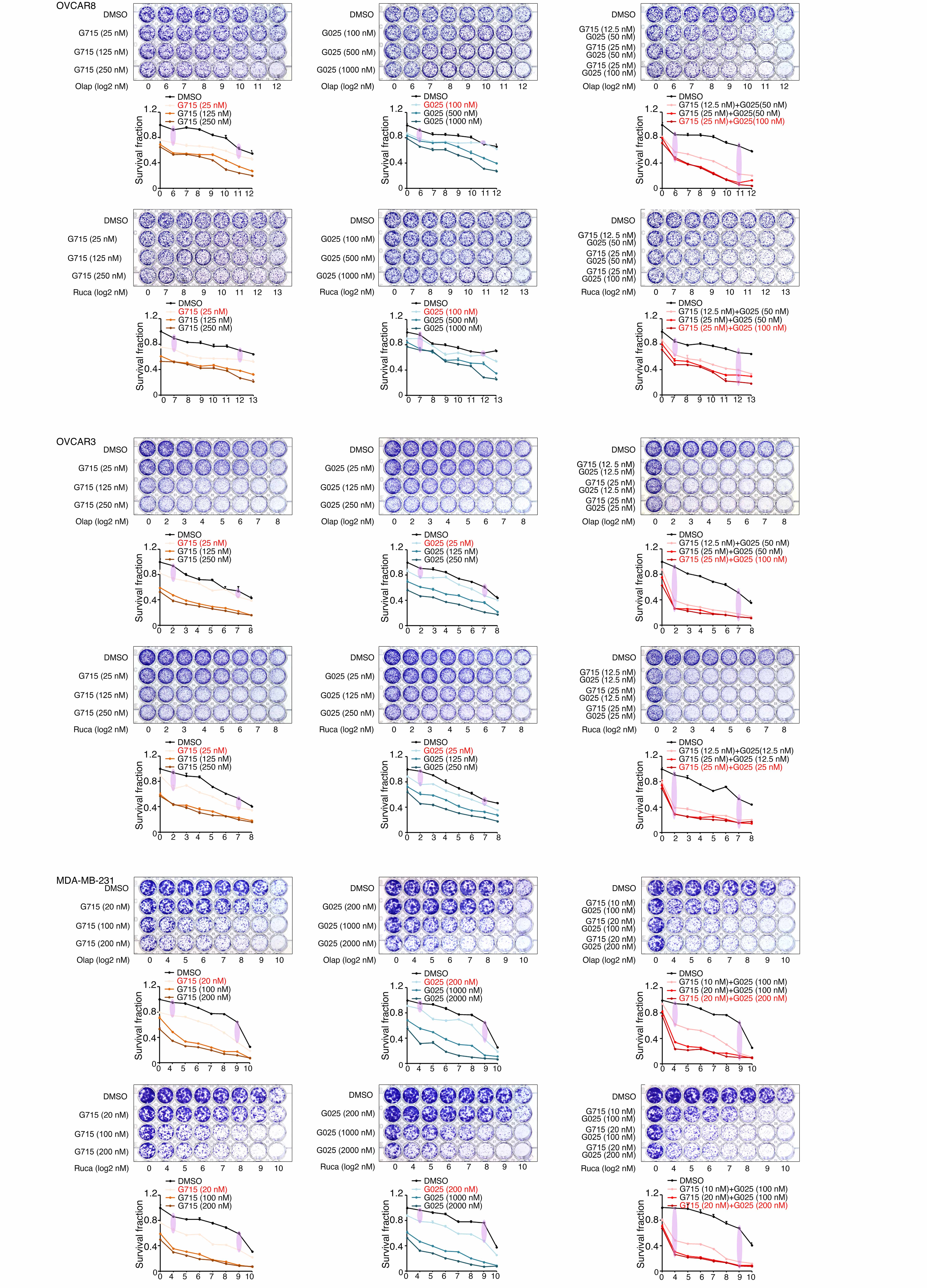

Supplement: Supplement 6 — Figure S6. PRMT1 and PRMT5 inhibition acts synergistically to enhance PARPi sensitivity in a panel of HGSOC and TNBC cell lines. [file media-6.jpg]
